# MNAT1 promotes proliferation and the chemo-resistance of osteosarcoma cell to cisplatin through regulating PI3K/Akt/mTOR pathway

**DOI:** 10.1186/s12885-020-07687-3

**Published:** 2020-12-03

**Authors:** Chensheng Qiu, Weiliang Su, Nana Shen, Xiaoying Qi, Xiaolin Wu, Kai Wang, Lin Li, Zhu Guo, Hao Tao, Guanrong Wang, Bohua Chen, Hongfei Xiang

**Affiliations:** 1 Department of Orthopedic Surgery, Affiliated Hospital of Qingdao University, Qingdao, 266000 China; 2 Department of Orthopedic Surgery, Qingdao Municipal Hospital (Group), Qingdao, 266011 China; 3 Department of Rehabilitation, Affiliated Hospital of Qingdao University, Qingdao, 266000 China; 4 Department of Gynaecology, Affiliated Hospital of Qingdao University, Qingdao, 266000 China; 5 Department of Operation Room, Affiliated Hospital of Qingdao University, Qingdao, 266000 China

**Keywords:** Osteosarcoma, MNAT1, Cisplatin

## Abstract

**Background:**

MNAT1 (menage a trois 1, MAT1), a cyclin-dependent kinase-activating kinase (CAK) complex, highly expressed in diverse cancers and was involved in cancer molecular pathogenesis. However, its deliverance profile and biological function in osteosarcoma (OS) remain unclear.

**Methods:**

The expression of MNAT1 in OS was detected by western blot (WB) and immunohistochemistry (IHC). The potential relationship between MNAT1 molecular level expression and OS clinical expectations were analyzed according to tissues microarray (TMA). Proliferation potential of OS cells was evaluated in vitro based on CCK8 and OS cells colony formation assays, while OS cells transwell and in situ tissue source wound healing assays were employed to analyze the OS cells invasion and migration ability in vitro. A nude mouse xenograft model was used to detect tumor growth in vivo. In addition, ordinary bioinformatics analysis and experimental correlation verification were performed to investigate the underlying regulation mechanism of OS by MNAT1.

**Results:**

In this research, we found and confirmed that MNAT1 was markedly over-expressed in OS tissue derived in situ, also, highly MNAT1 expression was closely associated with bad clinical expectations. Functional studies had shown that MNAT1 silencing could weaken the invasion, migration and proliferation of OS cells in vitro, and inhibit OS tumor growth in vivo. Mechanism study indicated that MNAT1 contributed to the progression of OS via the PI3K/Akt/mTOR pathway. We further verified that the MNAT1 was required in the regulation of OS chemo-sensitivity to cisplatin (DDP).

**Conclusions:**

Taken together, the data of the present study demonstrate a novel molecular mechanism of MNAT1 involved in the formation of DDP resistance of OS cells.

**Supplementary Information:**

The online version contains supplementary material available at 10.1186/s12885-020-07687-3.

## Background

Osteosarcoma (OS) is the more usual devastating bone cancer in the young people with a frequency increasing by 0.3% per year [1]. The morbidity rate of OS is relatively low (approximate 6% of all pediatric tumors) compared with other cancers, the five-year survival rate remains dismal due to the highly invasive potential [2]. Despite the great advances in comprehensive treatment of OS, prediction of early recurrence and metastasis is a big challenge for OS treatments [3, 4]. It is, hence, an improved understanding of the molecular mechanism of OS tumorigenesis may open up new avenues to develop novel therapeutic approaches for OS.

The third subunit besides CDK7 and Cyclin H in cyclin-dependent kinase-activating kinase (CAK) complex was originally determined to be MNAT1 [5]. Mounting evidence has revealed that MNAT1 play a pivotal part in regulation of biological characteristics of cancer cells. Recent report showed that MNAT1 was overexpressed in colorectal cancer, and its expression level was related to p53 ubiquitin-degradation and patient prognosis [6]. Furthermore, in leukemic cells, the enhancement of cell growth and metastasis is caused by the loss of inherent fragments of MNAT1 protein during particle production [7]. In breast cancer, expression of MNAT1 is bad expectations in Estrogen Receptor-Positive Breast Cancer [8]. In OS, MNAT1 play an important role in the lung metastasis of osteosarcoma [9]. MNAT1 promotes the malignant behaviors of osteosarcoma cells [10]. However, the underlying mechanism of MNAT1 in OS have not been well documented yet.

In this research, we found MNAT1 expression was increased in OS cells and tissues. Upregulation of MNAT1 expression was associated with bad clinical expectations of OS patients. In addition, downregulation of MNAT1 inhibited OS cell proliferation and invasion ability in vitro, suppressed and slowed down tumor growth in vivo. Moreover, we showed that MNAT1 overexpression enhanced cisplatin resistance, and that MNAT1 silencing restored the sensitivity and effective of OS cells to cisplatin, a chemotherapy drug, through regulation of the PI3K/Akt/mTOR pathway. Our study provided a novel insight for the function of MNAT1 in the sensitivity of OS to chemotherapy and the mechanism involved.

## Methods

### Clinical samples and cell lines

A total of 78 human OS samples and the corresponding non-tumor tissue samples were obtained from Affiliated Hospital of Qingdao University (Qingdao, China) between May 2009 and November 2018 (DQU cohort), after we obtained written informed consent according to an established protocol approved by the Ethics Committee of the Affiliated Hospital of Qingdao University. The diagnoses were confirmed by histological examination.

OS cell lines MG63, U2OS, Well5 and 143B were purchased from the American Type Culture Collection (ATCC, Manassas, USA), and Normal osteoblast cells HOBC and HFOB was acquired from the Cell Bank of the Chinese Academy of Sciences (Shanghai, China). The cell lines were cultured in Dulbecco’s modified Eagle’s medium (Gibco, Carlsbad, CA, USA) supplemented with 10% fetal bovine serum and 1% penicillin/streptomycin.

### Dataset acquisition and process

The independent OS microarray was extracted from The Cancer Genome Atlas (TCGA) database (http://gdc-portal.nci.nih.gov/). DESeq package in R language was used to compare the MNAT1 expression between OS and non-tumor samples in TCGA dataset. Functional and pathway enrichment analysis was performed based on TCGA OS database via Kyoto Encyclopedia of Genes and Genomes (KEGG) and Gene Set Enrichment Analysis (GSEA).

### Quantitative real-time PCR (qRT-PCR)

TRIzol was used to obtain the total RNA, which was refrigerated at − 80 °C. The biological spectrometer was then used to assess the RNA concentration. For another, the PrimerScript RT Master kit (Takara Biotechnology, Dalian, China) was used to do cDNA synthesis. The mRNA level of MNAT1 was detected by qRT-PCR with SYBRGreen PCR Master mix (Roche, Mannheim, Germany) on an ABI 7900 Real-Time PCR System (Applied Biosystems, Foster City, CA, USA). The relative fold-change in expression compared with control sample GAPDH was calculated using 2-^ΔΔ^Ct method. The specific primers used in this study were listed as following: MNAT1 (amplicon size: 160 bp, Tm: 60 °C), 5′-GGTTGCCCTCGGTGTAAGAC-3′ (forward) and 5′-AGTTGCTCTTTCTGAGTGGAGT-3′ (reverse); GAPDH (amplicon size: 231 bp, Tm: 60 °C), 5′- GAGAAGGCTGGGGCTCATTT − 3′ (forward) and 5′- AGTGATGGCATGGACTGTGG − 3′ (reverse);

### Tissue microarray (TMA) construction and immunohistochemistry (IHC)

The TMA were made from 78 OS tissues and matched nonneoplastic counterparts. A core (1.0-mm diameter) was punched from each OS tissue and arranged into the TMA blocks. For MNAT1 IHC analysis in TMA, MNAT1 staining was graded as negative (score 1+), weak (score 2+), moderate (score 3+) or strong (score 4+) for further nonparametric testing according to the percentage of cells staining positive and the staining intensity.

### Western blotting

Protein exacts were collected and lysed with RIPA lysis buffer. Equal amount of protein was loaded to sodium dodecyl sulfate-polyacrylamide gel electrophoresis (SDS-PAGE) and wet transferred to PVDF membranes. Membranes were incubated sequentially with specific primary antibodies and secondary antibodies. Primary antibodies against MNAT1 (Proteintech, 11,719–1-AP), β-tubulin (Abcam, ab210797), PI3K (Proteintech, 20,584–1-AP), Akt (Proteintech, 10,176–2-AP) and mTOR (Proteintech, 20,657–1-AP).

### Cell transfection

The MNAT1 siRNA and MNAT1 plasmid (si-MNAT1, MNAT1) were purchased from Ribobio (Guangzhou, China), a scramble oligonucleotide was referred to as negative control (si-NC). For transfections, 1 × 10^6^ cells (per well) were plated into a 6-well plate, and plasmids, siRNA targeting MNAT1 or negative control were transfected into the cells using Lipofectamine 2000 (Invitrogen, CA, USA) following the manufacturer’s instruction. The transfected cells were harvested after 48–72 h. The transfection efficiency was determined by qRT-PCR. The sequence of the siRNA and shRNA were listed as following: MNAT1-siRNA sequence: 5′-AAGACCACCAAATATCGGAAC-3′; MNAT1-shRNA sequence: 5′-CCGGCCTAGTCTAAGAGAATACAATCTCGAGATTGTATTCTCTTAGACTAGGTTTTT-3′.

### Lentivirus-mediated MNAT1 knockdown

For the stable knockdown of MNAT1, the annealed siRNA sequences were cloned into the LV-12 (pGLVH6-CMV-LUC-2A-Puro-U6-shRNA) vector to generate a MNAT1-shRNA lentivirus (shMNAT1) (Gene-Pharma, China). U2OS cells were transduced with the concentrated shMNAT1 or control virus.

### Cell proliferation and colony formation assays

When MNAT1 siRNA or MNAT1 plasmid was transfected into U2OS and 143B cells. 5 × 10^3^ cells per well were seeded into 96-well plates, with three wells used for each assayed group. Briefly, Cell numbers were evaluated over 5 days by CCK-8 Kit (Beyotime, China) method. The DNA synthesis rate was evaluated through Edu staining assay (Ribobio, Guangzhou, China), EdU positive cells were observed by using an Apollo and DAPI staining. For the colony formation assay, 1000 cells/well were plated into 6-well plates and routinely cultured for 14 days. The cells were subsequently fixed with 30% formaldehyde for 15 min and stained with 0.1% crystal violet. The number of colonies (containing more than 50 cells) was determined under an optical microscope. These experiments were in triplicate.

### Wound-healing assay

Wound-healing assays were carried out to determine the migration ability. U2OS and 143B cells expressing MNAT1 were seeded 24 h before the experiment. When the convergence degree of the cell reaches 90–100%, linear scratches are produced at the tip of the 200 μL pipette tip. Cells were observed using a light microscope (40× magnification) every 24 h. These experiments were in triplicate.

### Cell invasion assay

In vitro cell invasion was tested by Transwell assays. The logarithmic U2OS and 143B were collected and the number of cells was adjusted to 5 × 10^4^/ml, and then plated into the upper chamber in 200 μl completed medium. Six hundred microliter of completed medium containing 10% FBS was added into the lower chamber. After 24 h incubation, Transwell chamber with remaining cells was removed, and then the invaded cells were washed, fixed with 4% paraformaldehyde, and stained with crystal violet for 20 min. The number of invaded cells was counted in random non-overlapping field under a light microscope. The average number of cells in each field was counted.

### A xenograft tumor model

Six to eight-weeks-old male mice were used to construct xenograft tumor model. A total of 100 μl of a cell suspension containing 3 × 10^6^ U2OS cells stably transduced with a lentivirus expressing either sh-MNAT1 or sh-NC was subcutaneously injected into nude mice. The tumor volume was calculated by the formula: Tumor volume = (width)^2^ × length / 2. The study protocol was also approved by the Committee on the Use of Live Animals in Teaching and Research.

### Statistical analysis

All analyses were performed with GraphPad Prism V6 (Prism, USA). All experiments were repeated at least three times to calculate the mean and standard deviation (SD). The survival curves were calculated by Kaplan-Meier method. A * *p* < 0.05 is considered to be statistically significant.

## Results

### MNAT1 is highly expressed in OS cancer tissues and cell lines

**Fig. 1:**
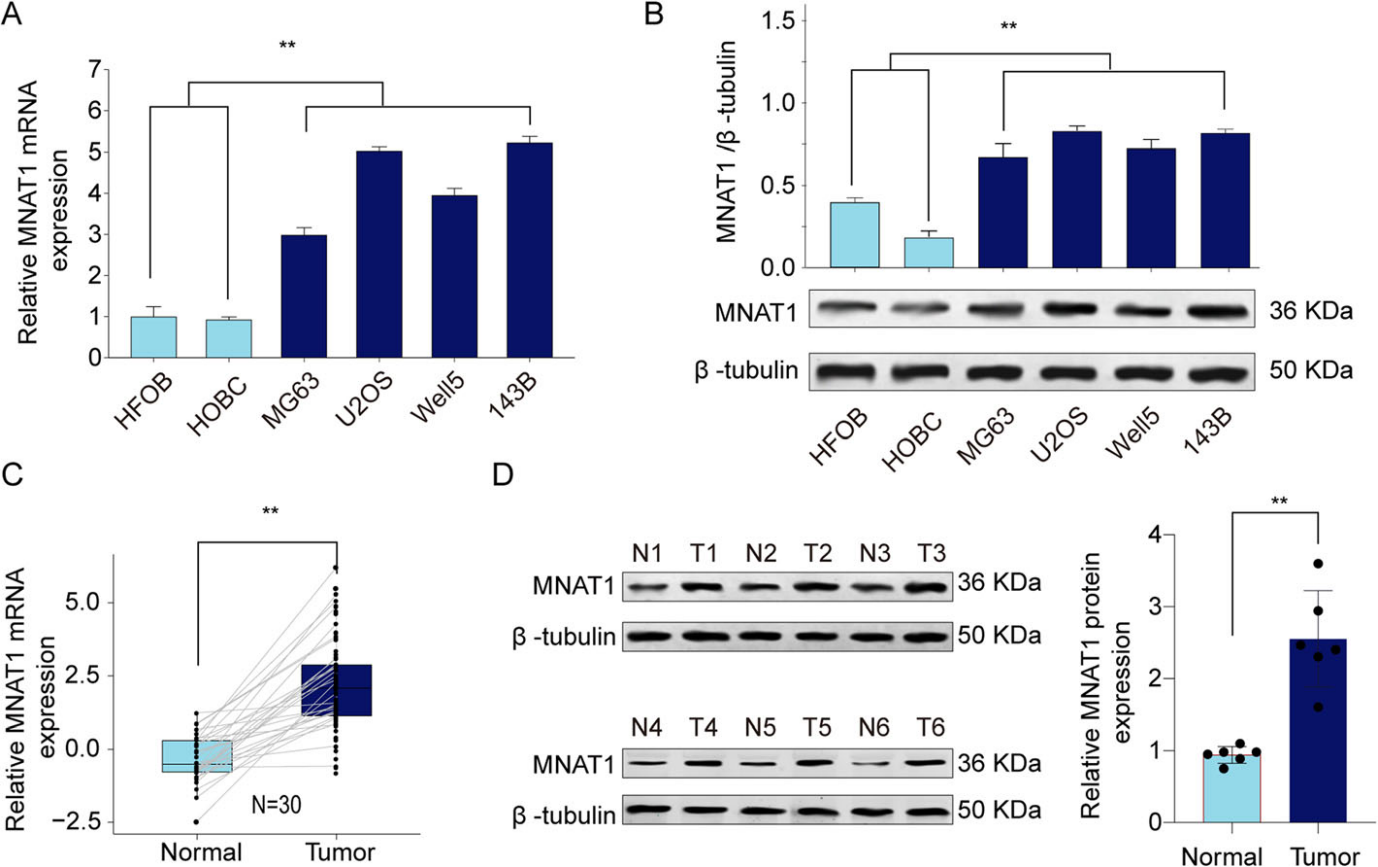
MNAT1 is upregulated in OS tissues and cell lines. **a** The mRNA expression of MNAT1 in OS cell lines (MG63, U2OS, Well5 and 143B) and control cells (HOBC and HFOB) was analyzed by qRT-PCR. **b** The protein expression of MNAT1 in OS cell lines and control cells. **c** The mRNA expression of MNAT1 in 30-paired OS tissues and adjacent normal tissues from affiliated hospital of qingdao university OS cohort was analyzed by qRT-PCR. **d** The protein expression of MNAT1 in 6-paired OS tissues (T) and adjacent normal tissues (N) was analyzed by western blot. *p < 0.05, **p < 0.01

With the purpose of determining the expression of MNAT1 in the OS, we evaluated the expression of MNAT1 in tissues from patients diagnosed with OS and several cell lines as representative of OS. Firstly, we found that the expression of MNAT1 was enhanced in MG63, U2OS, Well5 and 143B cells than that in control cell lines, the difference is significant (Fig. 1a, b). Simultaneously, we uncovered that mRNA levels of MNAT1 were up-regulated in 30-paired OS cases and surrounding tissue not invaded by the tumor (non-tumor tissue) (Fig. 1c). In addition, MNAT1 expression was remarkably higher in OS tumor tissue in situ than that in surrounding non-tumor tissues (Fig. 1d). These data showed that MNAT1 was overexpressed in OS cancer tissues and cells, thus might be engaged in OS cancer progression.

### MNAT1 overexpression correlates with bad expectations of OS patients

**Fig. 2:**
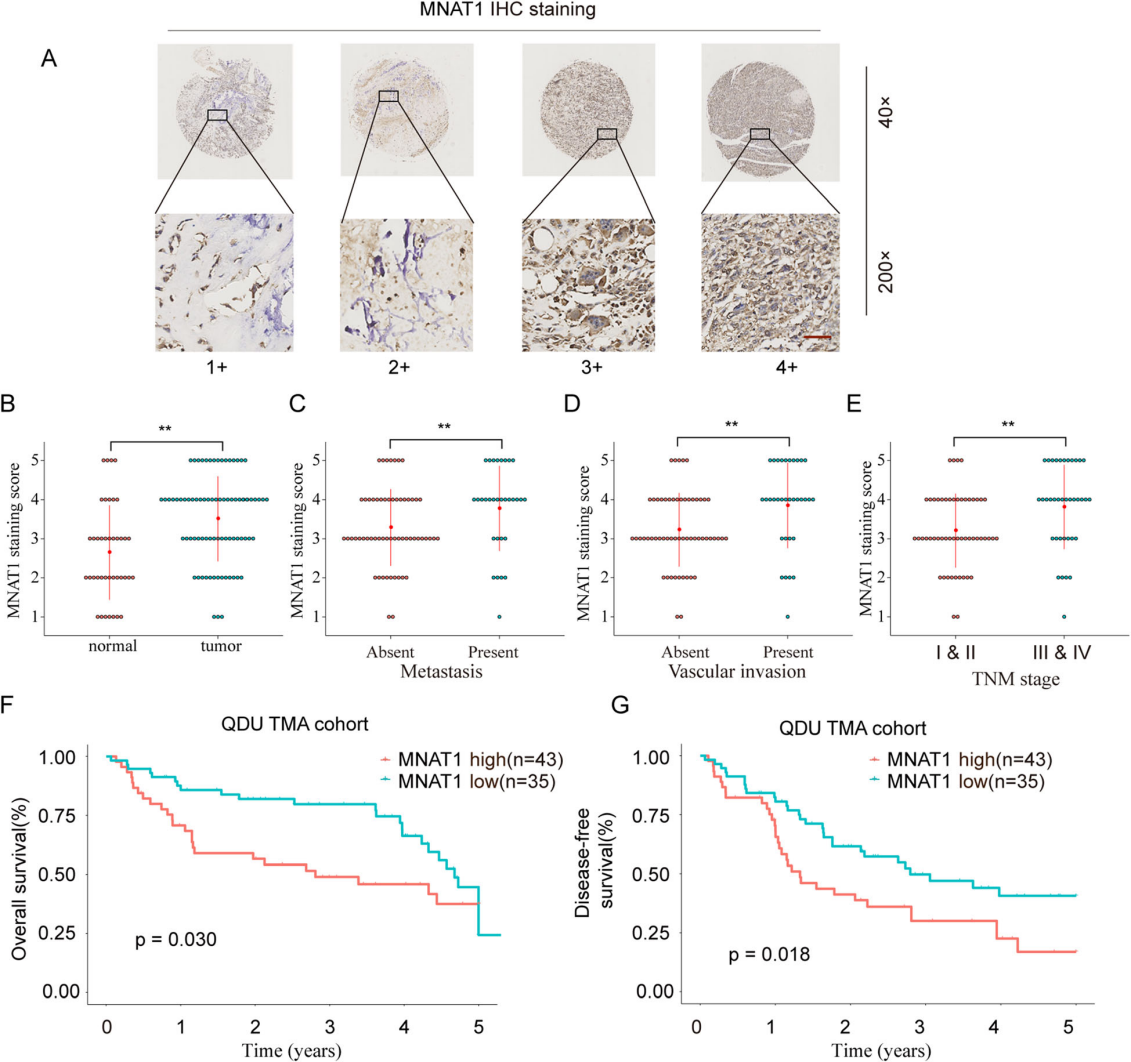
MNAT1 expression in OS tissues and its prognostic value. **a** Representative MNAT1 staining patterns via IHC assays. **b** MNAT1 expression was significantly higher in OS tissues (*n* = 78) compared with that in non-tumor tissues (*n* = 40). The correlation of MNAT1 expression level with distant metastasis (**c**), vascular invasion (**d**), and TNM stage (**e**). **f** and **g** The OS and DFS were analyzed by Kaplan-Meier analysis. *p < 0.05, **p < 0.01

Next, we analyzed the relationship between MNAT1 expressed characteristics and pathological features. MNAT1 protein expression was assessed on OS tissue microarrays (TMA) by immunohistochemistry (IHC) (Fig. 2a). Consistently, MNAT1 expression was significantly enhanced in OS tissues in comparison with that in normal control tissues (Fig. 2b). Moreover, we revealed that the expression of MNAT1 was positively correlated with distant transfer (Fig. 2c), vascular invasion (Fig. 2d), and TNM stage (Fig. 2e). Additionally, the survival of patients with OS in the MNAT1 low-expression group was apparently higher relative to that in the MNAT1 high-expression group (Fig. 2f, g). These results suggested that enhanced expression of MNAT1 may be closely associated with OS progression.

### MNAT1 knockdown suppresses OS cell proliferation and migration in vitro

**Fig. 3:**
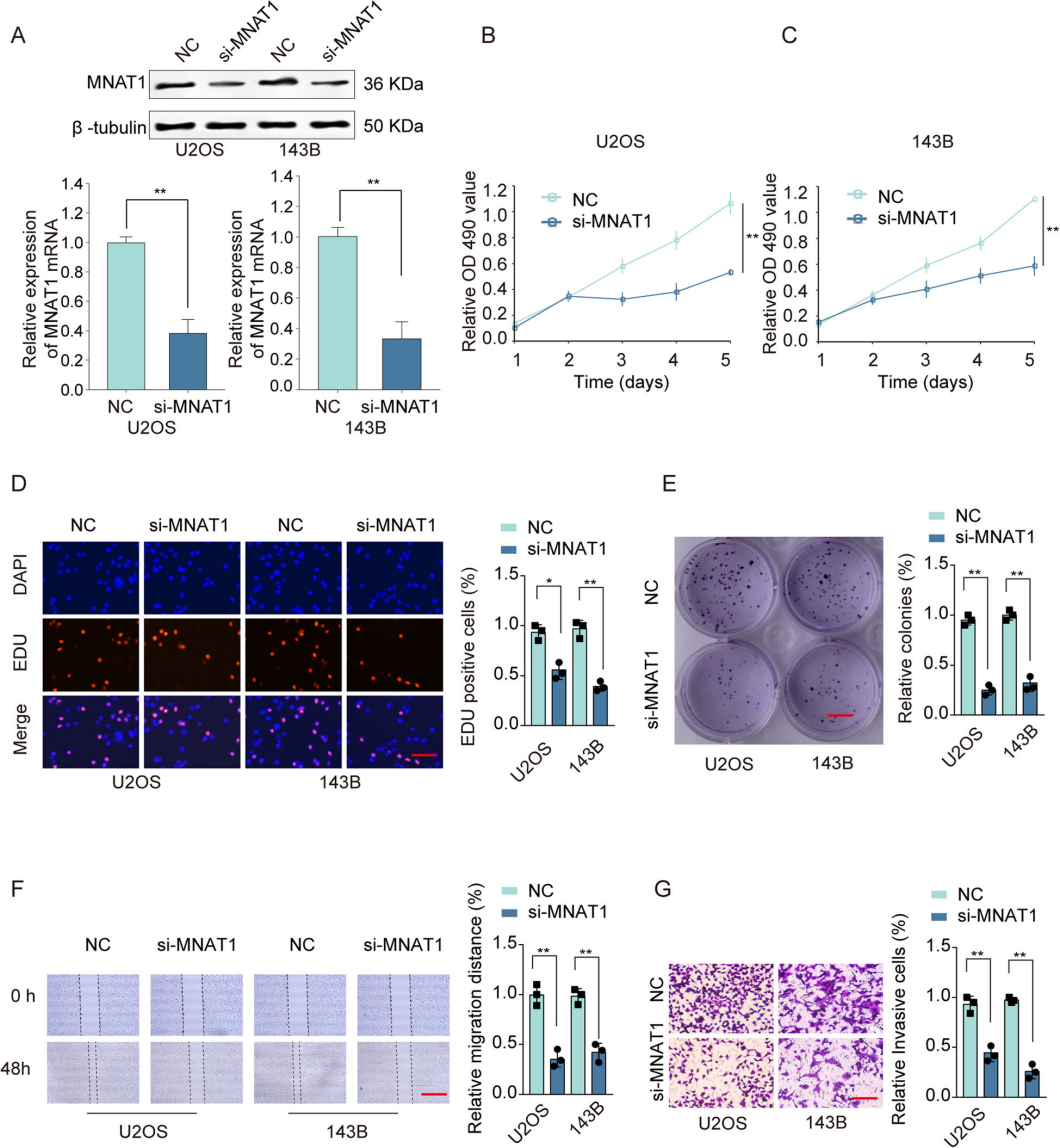
MNAT1 knockdown suppressed OS cell proliferation and migration. **a** The transfection efficiency of the MNAT1 siRNA was evaluated by qRT-PCR and western blot. **b** and **c** The changes in the proliferation of OS cells transfected with si-MNAT1 or NC were determined by the CCK-8 assay. **d** EdU staining assays (Scale bars, 50 μm) and **e** colony formation assays (Scale bars, 8 mm) were performed to determine the growth of U2OS and 143B cells. **f** Cell migration was assessed by wound-healing assay (Scale bars, 500 μm). **g** Cell invasion was assessed by transwell assay (Scale bars, 50 μm). *p < 0.05, **p < 0.01

To detect biological functions of the MNAT1 in OS cells, we performed functional experiments. We found that specific siRNAs targeting MNAT1 decreased the expression of MNAT1 after transfection of U2OS and 143B cells (Fig. 3a). Functionally, the results of CCK-8 experiments showed that knockdown of MNAT1 dramatically inhibited the cell growth compared with control group (Fig. 3b, c). In addition, knockdown of MNAT1 suppressed the DNA synthesis rate and colony formation compared with that in the negative control (Fig. 3d, e). In addition, the evaluation results of the migration and invasion potential of OS cells showed that the expression of MNAT1 had a significant inhibitory effect on the healing and invasion ability of cell wounds. (Fig. 3f, g). Thus, knockdown of MNAT1 impairs HCC cell aggressiveness in vitro.

### Enforced expression of MNAT1 promoted OS cell growth and invasion in vitro

**Fig. 4:**
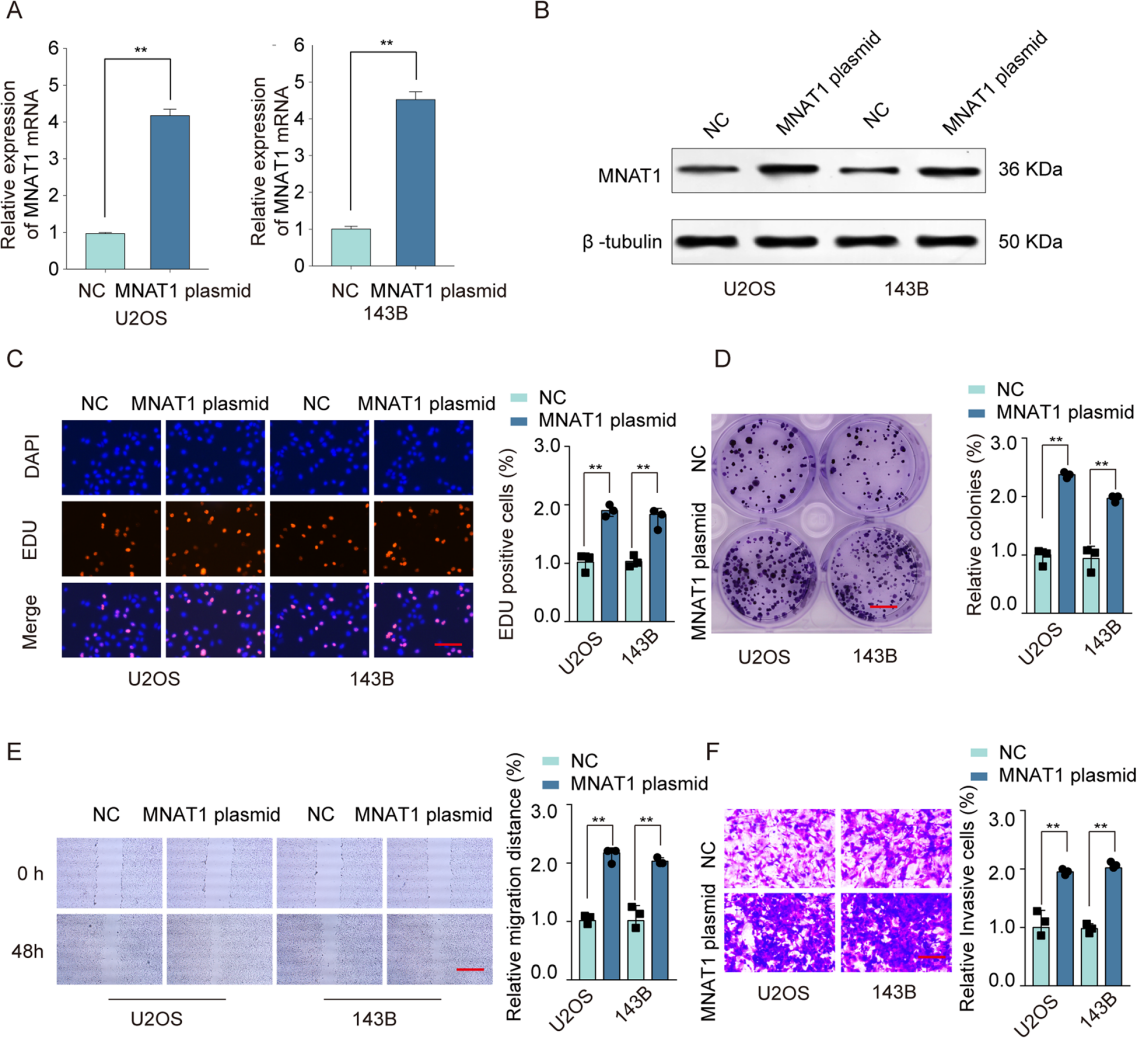
Ectopic overexpression of MNAT1 promotes OS cell proliferation and invasion in vitro*.* U2OS and 143B cells were transfected with MNAT1 plasmid or negative control (NC). **a** The mRNA expression of MNAT1 was examined by qRT-PCR. **b** The transfection efficiency was evaluated by western blot assay and EdU staining assays (Scale bars, 50 μm) (**c**) and colony formation assays (Scale bars, 8 mm) (**d**) were performed to determine the proliferation of U2OS and 143B cells. **e** Cell migration was assessed by wound-healing assay (Scale bars, 500 μm). **f** Cell invasion was assessed by transwell assay (Scale bars, 50 μm). *p < 0.05, **p < 0.01

Compared with negative control, MNAT1 plasmid significantly increased MNAT1 level in U2OS and 143B cells (Fig. 4a, b). Cell proliferation assays suggested that MNAT1 plasmid significantly promoted proliferation of U2OS and 143B cells compared with negative control (Fig. 4c, d). Migration and transwell assays confirmed the functional role of MNAT1 in OS cell metastasis ability, and the result showed that the migration and transwell ability of both the U2OS and 143B cells transfected with MNAT1 plasmid were significantly enhanced compared with the NC groups (Fig. 4e, f). These results indicated that MNAT1 may play an important role in OS cell growth and invasion.

### MNAT1 gene knockdown inhibit tumor proliferation in vivo

**Fig. 5:**
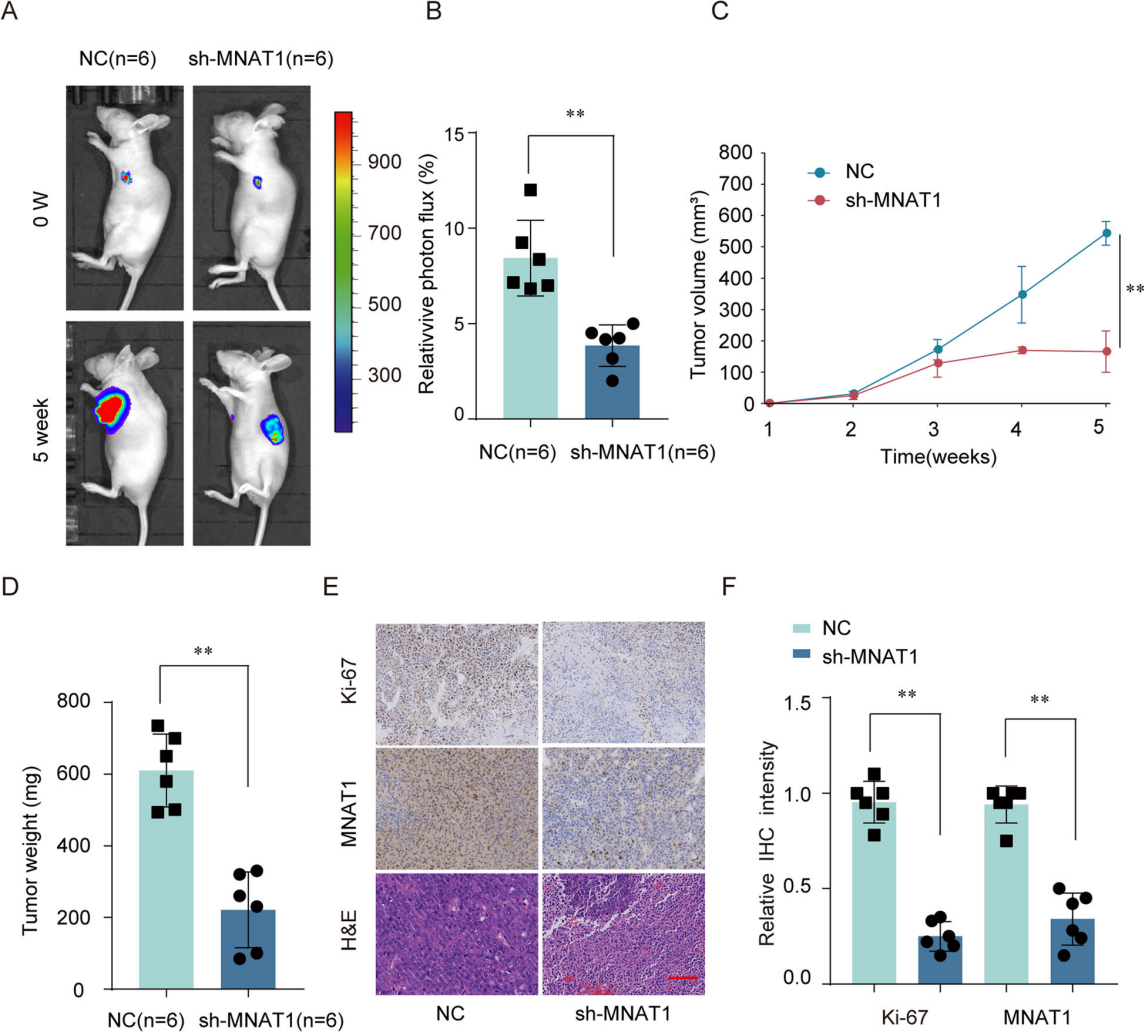
In vivo functional analysis of MNAT1 in OS. **a** The strength of the luciferase signal of xenograft tumor was decreased by MNAT1 knockdown. **b** The relative photon flux of OS tumor in nude mice of NC or sh- MNAT1 group was analyzed by a live imaging system to measure the luciferase signal. Comparison of tumor volume (**c**) and weight (**d**) in NC and sh-MNAT1 infected U2OS cells. **e** and **f** Representative IHC staining images and relative expression levels for MNAT1 and ki-67 in tumor sections from sh-MNAT1 group and NC group. *p < 0.05, **p < 0.01

Furthermore, we studied the biological effects of MNAT1 on OS proliferation in nude mice. MNAT1 stable knockdown U2OS cells or control cells were implanted subcutaneously and extramuscular to the right side of model mice. The results showed that the tumors developed from control cells had more mean luciferase signal than the xenograft tumors grown from cells knockdown MNAT1 (Fig. 5a, b). Moreover, knockdown of MNAT1 markedly reduced the tumor volume (Fig. 5c), with a much lower tumor tissue weight (Fig. 5d). In addition, IHC staining revealed that the relative expression of MNAT1 and proliferation marker Ki-67 were remarkably weaker in the tumor from MNAT1 knockdown group (Fig. 5e, f). Taken together, these results profound confirmation that MNAT1 knockdown significantly inhibits OS tumorigenesis.

### Activation of the PI3K/Akt/mTOR pathway was involved in the oncogenic functions of MNAT1 in OS

**Fig. 6:**
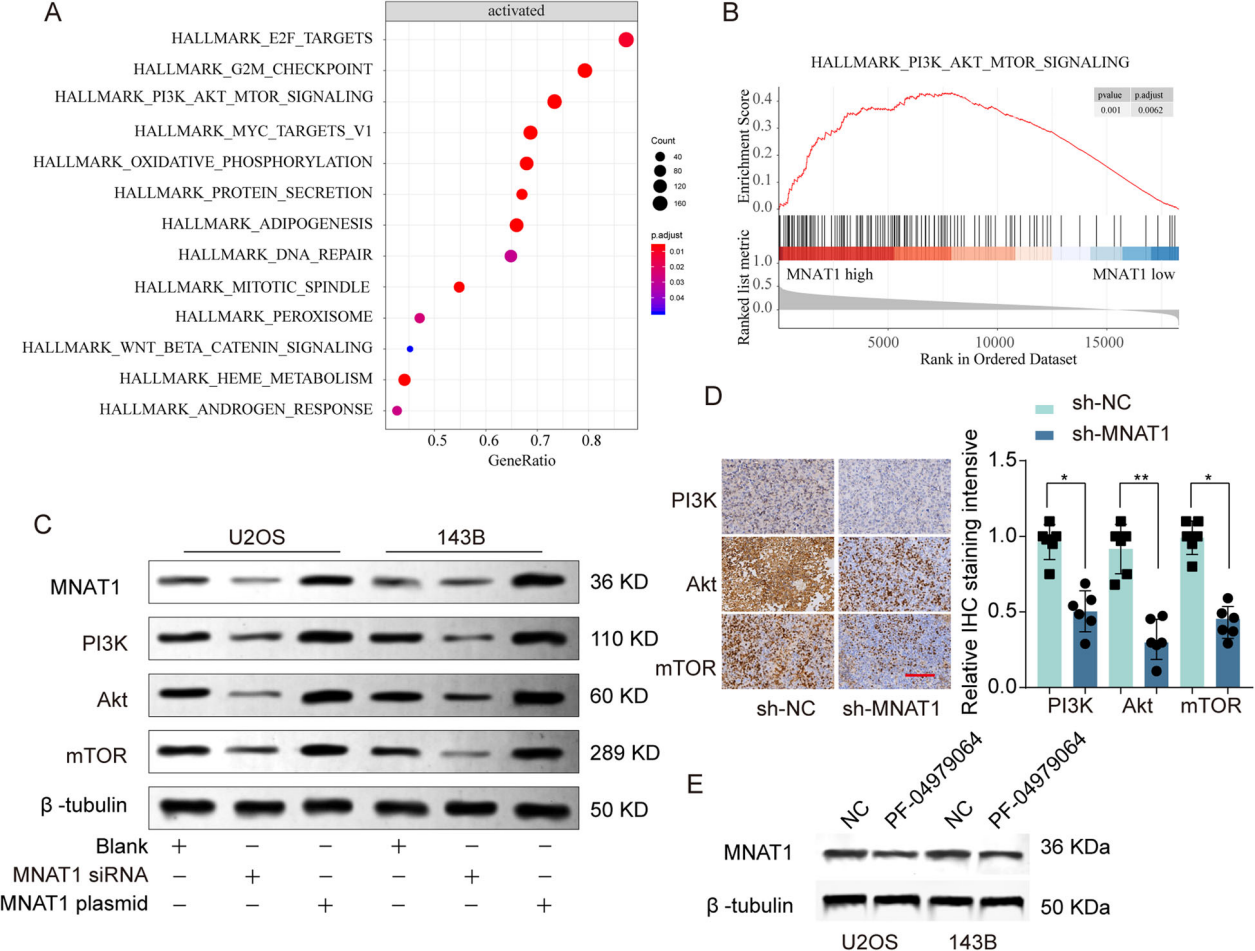
MNAT1 regulated PI3K/Akt/mTOR signaling pathway in OS. **a** KEGG analysis the different regulated signaling pathways in OS tumor compared with adjacent non-tumor tissues. **b** GSEA analysis the enrichment of signature genes in MNAT1 high or low group. **c** PI3K/Akt/mTOR pathway related proteins were analyzed by western blot. **d** IHC staining of PI3K, AKT and mTOR in xenograft tumor tissues from NC or sh-MNAT1 group. **e** The expression of MNAT1 was evaluated by western blot assay. * *p* < 0.05; ** *p* < 0.01

We used bioinformatics analysis that based on Cancer Genome Atlas (TCGA) OS database to analyze the mechanism of MNAT1 in OS. Kyoto Encyclopedia of Genes and Genomes (KEGG) and Gene Set Enrichment Analysis (GSEA) enrichment analysis suggested that PI3K/Akt/mTOR signaling pathway closely correlated with high MNAT1 expression (Fig. 6a, b, supplementary Figure S2). We repeated WB experiments using MNAT1 siRNA or MNAT1 plasmid in OS cells, in order to validate the results. It confirmed that the expression levels of PI3K/Akt/mTOR pathway associated proteins, such as PI3K, AKT and mTOR were sudden increased in MNAT1-overexpressed group while were decreased in MNAT1-silenced group (Fig. 6c). Additionally, IHC staining showed that expression of PI3K, AKT and mTOR was decreased in the MNAT1-knockdown xenograft tumor tissues (Fig. 6d). In order to verify the effect of PI3K signaling on the activity of MNAT1, we repeated the western blot experiment using PI3K inhibitor PF-04979064. Compared with negative control, PF-04979064 significantly decreased MNAT1 level in U2OS and 143B cells (Fig. 6e). These results suggest that MNAT1 might regulate the activation of PI3K/Akt/mTOR signaling pathway in OS cancer cells.

### MNAT1 regulated OS chemo-sensitivity to DDP-based therapy

**Fig. 7:**
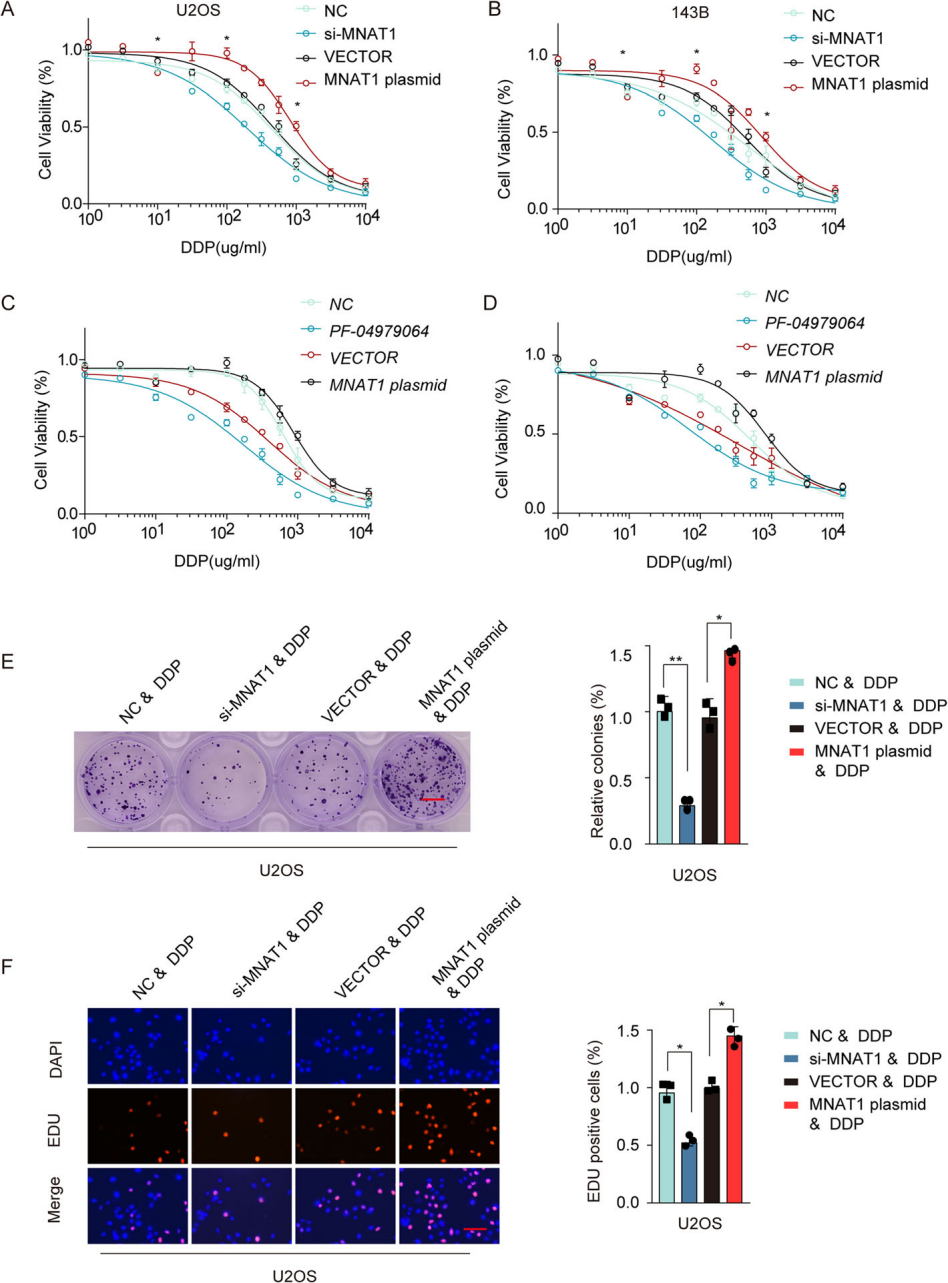
MNAT1 regulated OS chemo-sensitivity to DDP-based therapy. U2OS and 143B cells were transfected with si-NC, si-MNAT1, VECTOR or MNAT1 plasmid. After 48 h cells were treated with different concentration of DDP. **a** and **b** Cell viability analysis of U2OS and 143B cells treated with si-MNAT1 or MNAT1 plasmid and different concentrations of DDP in comparison with the negative control. **c** and **d** Cell viability analysis of U2OS and 143B cells treated with PI3K inhibitor PF-04979064 or MNAT1 plasmid and different concentrations of DDP in comparison with the negative control. **e** and **f** Colony formation (Scale bars, 8 mm) and EdU assay (Scale bars, 50 μm) performed on U2OS cells treated with si-MNAT1 or MNAT1 plasmid and different concentration of DDP in comparison with the negative control

To investigate whether MNAT1 was involved in the chemosensitivity of OS to DDP, we used a gain or loss-of-function approach in U2OS and 143B cells. As shown in Fig. 7a and b, the cell viability was suppressed by DDP treatment, the inhibitory effect on cell viability increased along with the increase of concentration. MNAT1 overexpression significantly suppressed the sensitivity of U2OS and 143B cells to DDP. Meanwhile, the cell viability was repressed after si-MNAT1 transfection under DDP treatment. However, treatment with the PI3K inhibitor PF-04979064 promoted the sensitivity of U2OS and 143B cells to DDP (Fig. 7c, d). Consistently, the results of Colony formation and EdU assay shown that MNAT1 silencing remarkably impeded the OS cells proliferation. While, the overexpression of MNAT1 showed the opposite results (Fig. 7e, f). These results collectively support that the increasing expression of MNAT1 might inhibit drug sensitivity to DDP in OS cancer cells.

## Discussion

Although in-depth knowledge has been made in the diagnosis and treatment strategies of OS, due to its delayed diagnosis and lack of effective treatment strategies, its mortality rate remains high [3, 11]. Therefore, it is essential to have a thorough understanding of the underlying mechanisms of OS carcinogenesis in order to develop new OS treatment strategies.

Tumor is caused by the accumulation of multiple oncogenes and the inactivation of tumor suppressor genes. This research confirm that MNAT1 is a neoteric oncogene in OS clinical expectations. In this research, we revealed that MNAT1 was upregulated in OS cancer tissues and cell lines (Fig. 1). In order to test and verify the effect of MNAT1 on the activity of CAK, we performed WB experiments using MNAT1 siRNA or MNAT1 plasmid in OS cells. We proved that the expression levels of CDK7 and Cyclin H were dramatically increased in MNAT1-overexpressed group while were decreased in MNAT1-silenced group (supplementary Figure S1). More importantly, the high level of MNAT1 generally indicates the high metastasis of tumor, vascular invasion and advanced TNM stage. In addition, high expression of MNAT1 was positively associated with prognosis survival rates of OS patients (Fig. 2). Functional assays revealed that MNAT1 knockdown significantly suppressed the proliferation and invasion of OS cells in vitro (Fig. 3). While, the overexpression of MNAT1 showed the opposite results (Fig. 4). In vivo experiments further demonstrated the positive effect of MNAT1 on OS cell growth (Fig. 5). Consistent with our data, MNAT1 was reported to mediate p53 ubiquitin-degradation and promote colorectal cancer malignance [6]. Moreover, in breast cancer, elevated expression of MNAT1 predicted poor prognosis and served as an oncogene [8]. Recent literature has documented that MNAT1 serves as a promoter to malignant behavior and lung metastasis of osteosarcoma. However, biological function and its molecular regulation mechanism of MNAT1 in OS remain largely unknown. We need more studies to dissect the precise role of MNAT1 in OS development.

To further investigate mechanism of MNAT1 on progression of OS, previously described bioinformatics and mechanism studies indicated that PI3K/Akt/mTOR pathway was extremely relevant to expression of MNAT1 (Fig. 6a and b). A large number of research have confirmed that PI3K/Akt/mTOR signaling plays a vital role in tumorigenesis [12–14], especially OS [15, 16]. Due to hyperactivation of the PI3K/Akt/mTOR pathway could lead to aberrant cell growth and tumor invasion [17, 18], thus investigated the mechanism of hyperactivation of this signaling pathway was the key to find new targets for tumor therapy. Our study demonstrated that suppression of MNAT1 could significantly inhibit activation of PI3K/Akt/mTOR pathway, and decrease expression of PI3K/Akt/mTOR pathway associated genes, including PI3K, Akt and mTOR (Fig. 6c and d). Compared with negative control, PI3K inhibitor PF-04979064 decreased MNAT1 level in U2OS and 143B cells (Fig. 6e). Based on these, our findings indicate that MNAT1 contributes to progression of OS through regulating the PI3K/Akt/mTOR signaling.

Previous studies reported that OS is prone to multidrug resistance [19–21]. However, whether MNAT1 is involved in the mechanism of OS drug resistance is still insufficient, which is one of the future research directions. In the current study, OS cell viability was inhibited by DDP treatment, while suppressed by si-MNAT1 transfection and promoted by MNAT1 overexpression (Fig. 7), suggesting that MNAT1 might regulate the chemo-sensitivity of OS cell through certain pathway. According to our studies, MNAT1 has been regarded as an oncogene through activating PI3K/Akt/mTOR pathway in OS (Fig. 6). In osteosarcoma, Rh2 has an anticancer effect on U20S cells by regulating PI3K/Akt/mTOR signaling pathway [22]. MicroRNA-22 mediates the cisplatin resistance of osteosarcoma cells by inhibiting autophagy via the PI3K/Akt/mTOR pathway [23]. The increase of cisplatin resistance in osteosarcoma can be achieved by inducing PI3K / Akt / mTOR signaling pathway by OIP5-AS1 [24]. However, it still remained unclear whether MNAT1 affected the chemo-sensitivity of OS by regulating PI3K/Akt/mTOR pathway. To investigate the mechanism by which MNAT1 exerts its function in OS sensitivity to DDP could provide a solid theoretical basis for further clinical application.

## Conclusions

MNAT1 was upregulated and functioned as an oncogene through activating PI3K/Akt/mTOR pathway in OS. Knockdown of MNAT1 inhibited OS proliferation and invasion both in vitro and in vivo*.* Moreover, MNAT1 overexpression contributed to the enhanced malignancy in OS cells including high proliferation, high invasion, and enhanced DDP chemoresistance. Our results suggest a profound insight into the development of OS, and provide a potential therapeutic target for the treatment of OS.

## Supplementary Information

**Additional file 1:** **Figure S1.** MNAT1 regulated the activity of CAK. U2OS and 143B cells treated with si-MNAT1 or MNAT1 plasmid. Western blot analysis of CDK7 and Cyclin H proteins in U2OS and 143B cells.

**Additional file 2:** **Figure S2** Mechanism study of OS by MNAT1. (A, B) GSEA analysis the enrichment of pathways between MNAT1 high group and low group.

## Data Availability

The data sets are available from the corresponding author on reasonable request.

## Abbreviations

MNAT1: Menage a trois 1
OS: Osteosarcoma
qRT-PCR: Quantitative RT-PCR
DDP: Cisplatin
TMA: Tissue microarrays
IHC: Immunohistochemistry
TCGA: Cancer Genome Atlas
KEGG: Kyoto Encyclopedia of Genes and Genomes
GSEA: Gene Set Enrichment Analysis
CAK: Cyclin-dependent kinase-activating kinase
