# Supplementary figures and images for: MNAT1 promotes proliferation and the chemo-resistance of osteosarcoma cell to cisplatin through regulating PI3K/Akt/mTOR pathway
Source: BMC Cancer. 2020 Dec 3;20:1187. doi: 10.1186/s12885-020-07687-3 (PMC7713032; doi:10.1186/s12885-020-07687-3)

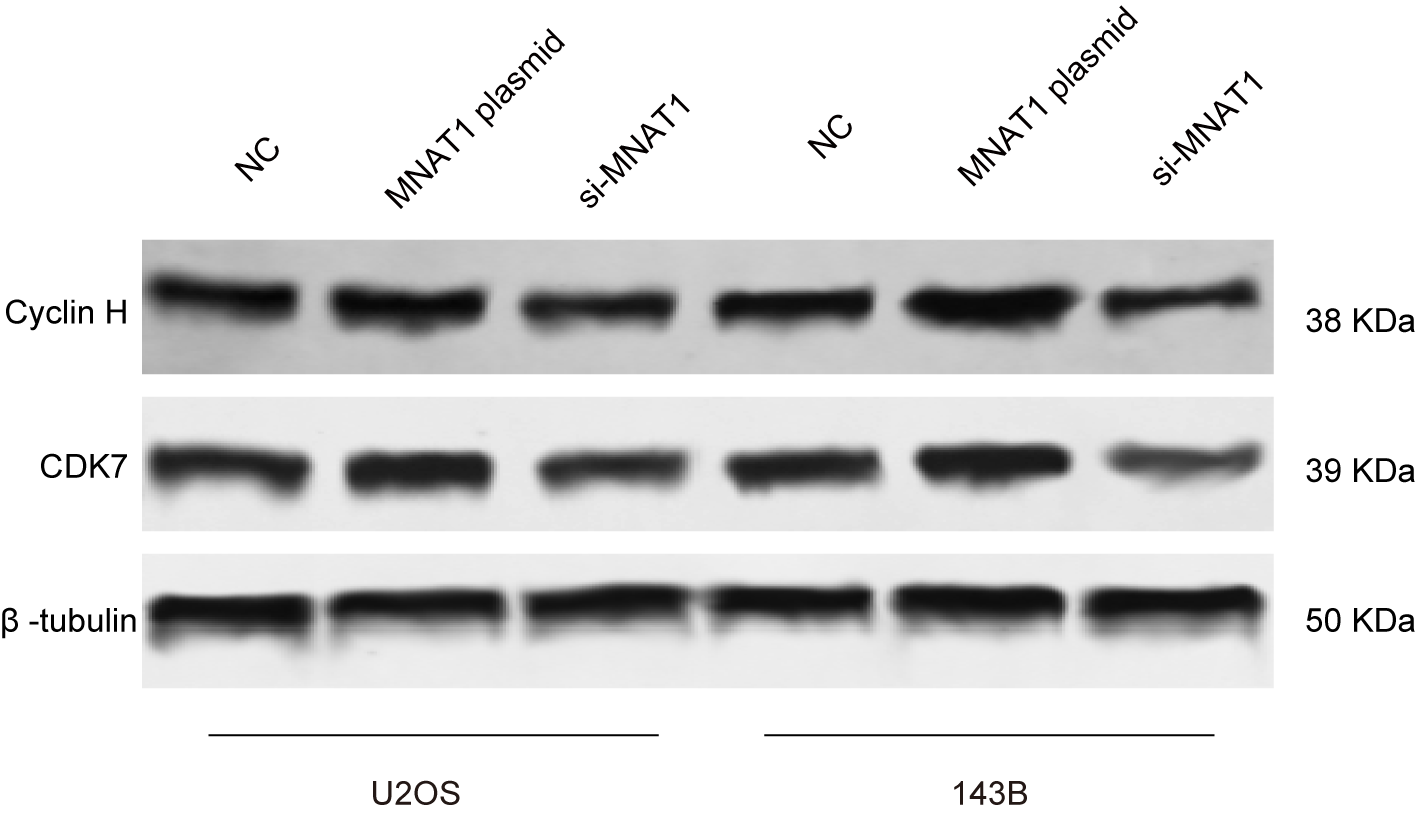

Supplement: Supplementary file 1 — Additional file 1: Figure S1. MNAT1 regulated the activity of CAK. U2OS and 143B cells treated with si-MNAT1 or MNAT1 plasmid. Western blot analysis of CDK7 and Cyclin H proteins in U2OS and 143B cells. [file 12885_2020_7687_MOESM1_ESM.tif]

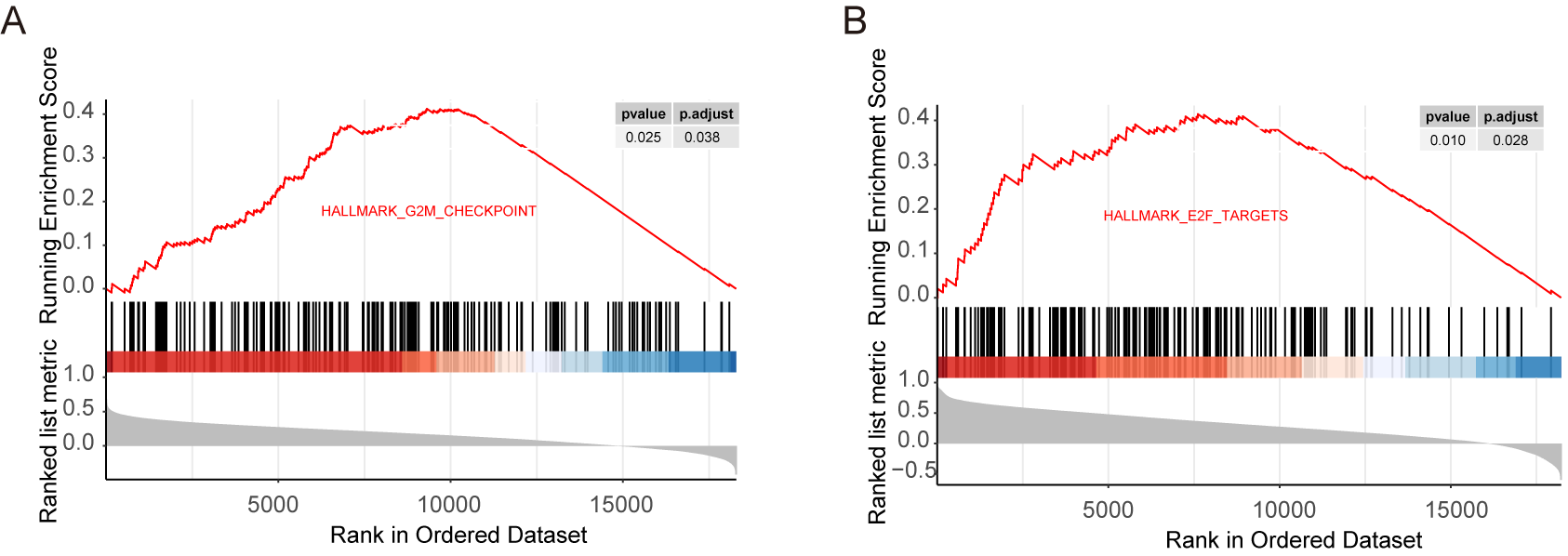

Supplement: Supplementary file 2 — Additional file 2: Figure S2 Mechanism study of OS by MNAT1. (A, B) GSEA analysis the enrichment of pathways between MNAT1 high group and low group. [file 12885_2020_7687_MOESM2_ESM.tif]
